# Extinction Events Can Accelerate Evolution

**DOI:** 10.1371/journal.pone.0132886

**Published:** 2015-08-12

**Authors:** Joel Lehman, Risto Miikkulainen

**Affiliations:** 1 Center for Computer Games Research, IT University of Copenhagen, Copenhagen, Denmark; 2 Department of Computer Science, University of Texas at Austin, Austin, Texas, United States of America; Saint Mary’s University, CANADA

## Abstract

Extinction events impact the trajectory of biological evolution significantly. They are often viewed as upheavals to the evolutionary process. In contrast, this paper supports the hypothesis that although they are unpredictably destructive, extinction events may in the long term *accelerate* evolution by increasing evolvability. In particular, if extinction events extinguish indiscriminately many ways of life, indirectly they may select for the ability to expand rapidly through vacated niches. Lineages with such an ability are more likely to persist through multiple extinctions. Lending computational support for this hypothesis, this paper shows how increased evolvability will result from simulated extinction events in two computational models of evolved behavior. The conclusion is that although they are destructive in the short term, extinction events may make evolution more prolific in the long term.

## Introduction

Extinction events exert a powerful influence on evolution [1–4]. For example, the Cretaceous-Paleogene extinction event is believed to have displaced non-avian dinosaurs with mammals in many ecological niches [1, 3], potentiating the later evolution of humans. However, an interesting question is whether such extinction events result in any consistent evolutionary outcomes, or whether they instead act as unpredictable filters that cause upheaval in evolution while producing little regular effects [5].

It is plausible that no general adaptation can protect an organism or species from an extinction event. Such events are often idiosyncratic and impose selection criteria radically different from those representative of its evolutionary history as a whole [1, 4]. In this view, extinction events may *disturb* evolution by stochastically discarding a large proportion of accumulated genetic material [4, 5]. In such regimes, extinction events constitute an abiotic influence that emphasizes historical contingency over biotic competition between organisms [6, 7].

However, while extinction events are unpredictable and destructive, they may also induce evolutionary regularities and thus *accelerate* evolution [8]. In particular, this paper probes a potential link between extinction events and the biological property of *evolvability*, i.e. the potential of an organism’s lineage to further evolve [9, 10]. While this connection has been explored in previous work [8], the model presented in this paper is more general, i.e. it does not depend upon direct biotic competition. the model also demonstrates that it is possible to harness this mechanism to for enhance practical engineering applications of evolutionary algorithms.

The particular definition of evolvability adopted in this paper follows a mainstream conception of evolvability as phenotypic variability [9–12]; that is, the capacity of an organism’s lineage to generate novel phenotypic traits. Although there exist alternative definitions that focus on different or overlapping aspects of evolvability [11], this definition captures a significant part of what enables some lineages to adapt more quickly than others.

The overall hypothesis is that repeated extinction events may result in increasing evolvability. By creating a survival bottleneck dependent upon unpredictable phenotypic traits [5], extinction events may indirectly select for lineages that can diversify quickly across the space of such phenotypes.

This idea is related to adaptive radiation: If extinction events empty a significant proportion of inhabited niches at unpredictable intervals, an advantageous strategy is to diversify through as many niches as possible. That way, a lineage increases its chances of occupying niches that through chance are not extinguished by extinction events. Thus, if radiating through niches generally requires modifying phenotypic traits, then this process of stochastic emptying and re-filling of ecological niches may select indirectly for the ability to radiate quickly, i.e. higher evolvability.

A complementary perspective is that ecological niches may become saturated, leading to evolutionary stagnation that extinction events disrupt. If increasing evolvability is enabled by diversifying and radiating through unoccupied niches [13] but such unoccupied niches are quickly saturated [14, 15], then this force may remain blunted. However, extinction events counteract such stagnation by emptying occupied ecological niches to thereby enable further radiations.

Like evolvability, ecological niches can be defined in alternative ways [16, 17], and it is thus important to clarify how the term is used in this paper. Because the extinction event hypothesis paper is tested computationally, niche in this paper refers to a computational concept. Abstracting the idea that niches support different ways of life that depend upon different ecological resources, computational niches are solutions that depend upon different computational representations. This kind of definition is often adopted within the fields of artificial life [18] and evolutionary computation [19], which build on computational abstractions of evolutionary concepts. The definition is instantiated separately for each of computational models used, as will be explained further below.

To test the hypothesis, experiments with two different computational models are conducted. In an abstract model, the results demonstrate that an evolutionary process that includes extinction events tends to generate more evolvable organisms than does an identical process without such events. In a concrete model, these results are put to work in evolutionary robotics (ER; [20]), demonstrating how the underlying mechanism may enhance evolutionary algorithms to solve practical engineering problems. The conclusion is that extinction events may provide an important creative force in both biological and artificial evolution.

## Experiments

The two subsections below describe the experiments with the two computational models used to test the hypothesis.

### The Abstract model

The first experiment investigates whether extinction events increase evolvability in an abstract model. More specifically, this experiment extends the abstract *limited-capacity niche* model of Lehman and Stanley [13].

In this minimal model, each organism is represented by two variables: niche and evolvability. First, niches are abstracted as locations in two-dimensional space. An organism’s niche is thus a two-dimensional coordinate in a discrete toroidal grid. The idea is to abstract coarsely the concept of a connected space of different ways of life. Mutation perturbs this niche by shifting it one unit in either dimension. This spatial structure models the constraint that transitions are more probable between niches with similar resources and environments. Overall, the motivation is to provide an abstract space through which organisms populate and radiate, inspired by the concept of adaptive radiation through ecological niches.

Second, an organism’s evolvability (i.e. its lineage’s capacity to generate novel phenotypes) is represented as the probability that its offspring will shift niches through mutation. This representation assumes that novel phenotypic traits are necessary for the organism to live in a new niche [21, 22], and thus that phenotypic variability will lead to more diversity [9]. Codifying the assumption that evolvability is heritable, an organism’s shift probability is inherited by its offspring. It mutates through small uniform perturbation at an infrequent *fixed* rate; in this way, offspring have the potential to become more or less evolvable than their parents.

A simple evolutionary process acts on a population of such organisms. At each generation an organism has two offspring, resulting in geometric growth in population size. However, to model limited resources in natural evolution (which bounds growth), niches support only a limited number of individuals. Additional organisms beyond that limit are discarded. To seed evolution, a single ancestral organism is placed into an initial niche at the center of the space of niches.

As shown in prior work [13], indirect pressure to expand through niches results in increasing evolvability over evolution. Lineages of more evolvable organisms radiate more quickly through the space of niches and end up populating more of them. However, because there are a limited amount of niches in the model, evolvability grows only until the capacity of all the niches becomes fully saturated. At that point, evolvability changes only through drift, which on average will cause evolvability to stagnate. Analogously in nature, unoccupied niches tend to become rare over time [14], blunting evolvability. Extinction events thus offer a way to facilitate further radiations and evolvability.

To test this hypothesis, the abstract model was modified to include extinction events. At arbitrary intervals, nearly all populated niches are extinguished, i.e. all individuals in them removed. More specifically, extinction events are modeled as pulsed (i.e. there is insufficient time to adapt to the evolutionary shock [23]) and wanton [5]. Extinctions are also severe: Only five niches (chosen randomly) survive immediate extinction (although the model is not sensitive to severity, as highlighted by S1 Fig). Following an extinction event, organisms from the five remaining niches then repopulate the model over time.

Five different conditions are compared in this experiment:

- In the *Control* condition there are no extinction events; the model remains the same as the abstract limited-capacity niche model of Lehman and Stanley [13].
- In the *Extinction 1000*, *Extinction 2000*, and *Extinction 4000* conditions extinction events occur at regular intervals of 1000, 2000, and 4000 generations, respectively. The motivation for these conditions is to explore how changing the frequency of extinctions impacts evolvability.
- In the *Extinction Random* condition extinction events occur at random intervals, with each interval chosen separately from a distribution of uniform probability between 1000 and 4000 generations. The idea is to remove the simplification of regular and predictable extinction events.

**Fig 1:**
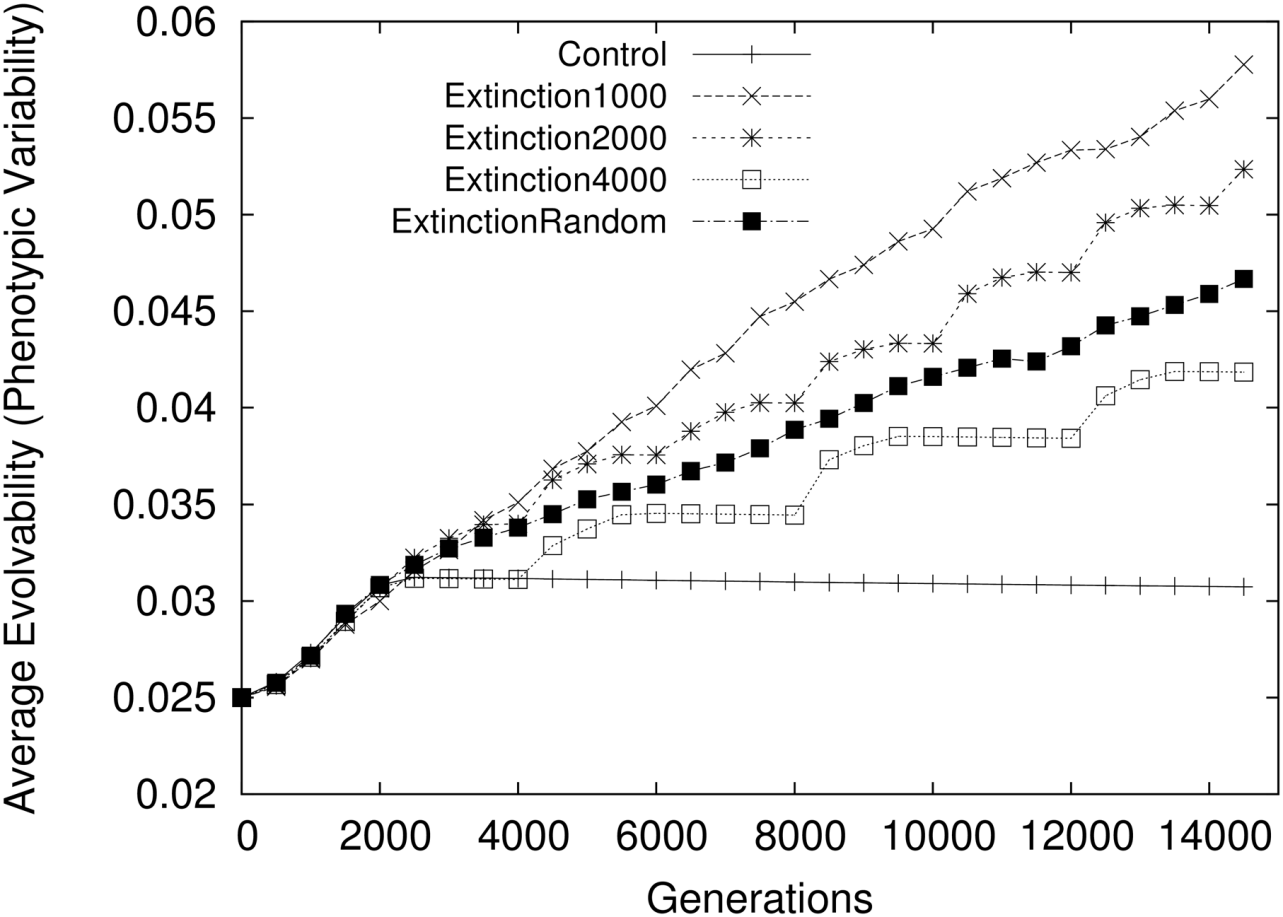
Evolvability in the abstract model. The average evolvability of individuals in the population, averaged over 50 independent runs is shown for the Control condition (with no extinction events), the Extinction 1000, 2000, and 4000 conditions (with extinctions regularly spaced respectively 1000, 2000,and 4000 generations, and the Extinction Random condition (where extinctions are spaced randomly with interval lengths chosen uniformly between 1000 and 4000 generations). In the Control condition there is no significant increase in evolvability following the initial 2000 generations, during which all niches become saturated (Mann-Whitney U-test;*p* > 0.05). However in each of the Extinction conditions, evolvability is significantly higher by the end of evolution (Mann-Whitney U-test;*p* < 0.05). The final average evolvability for each condition is statistically significantly different from each other condition (Mann-Whitney U-test;*p* < 0.05 for each pair-wise comparison of conditions’ final average evolvability). The conclusion is that the more frequent the extinction events, the more evolvable the final population.

**Fig 2:**
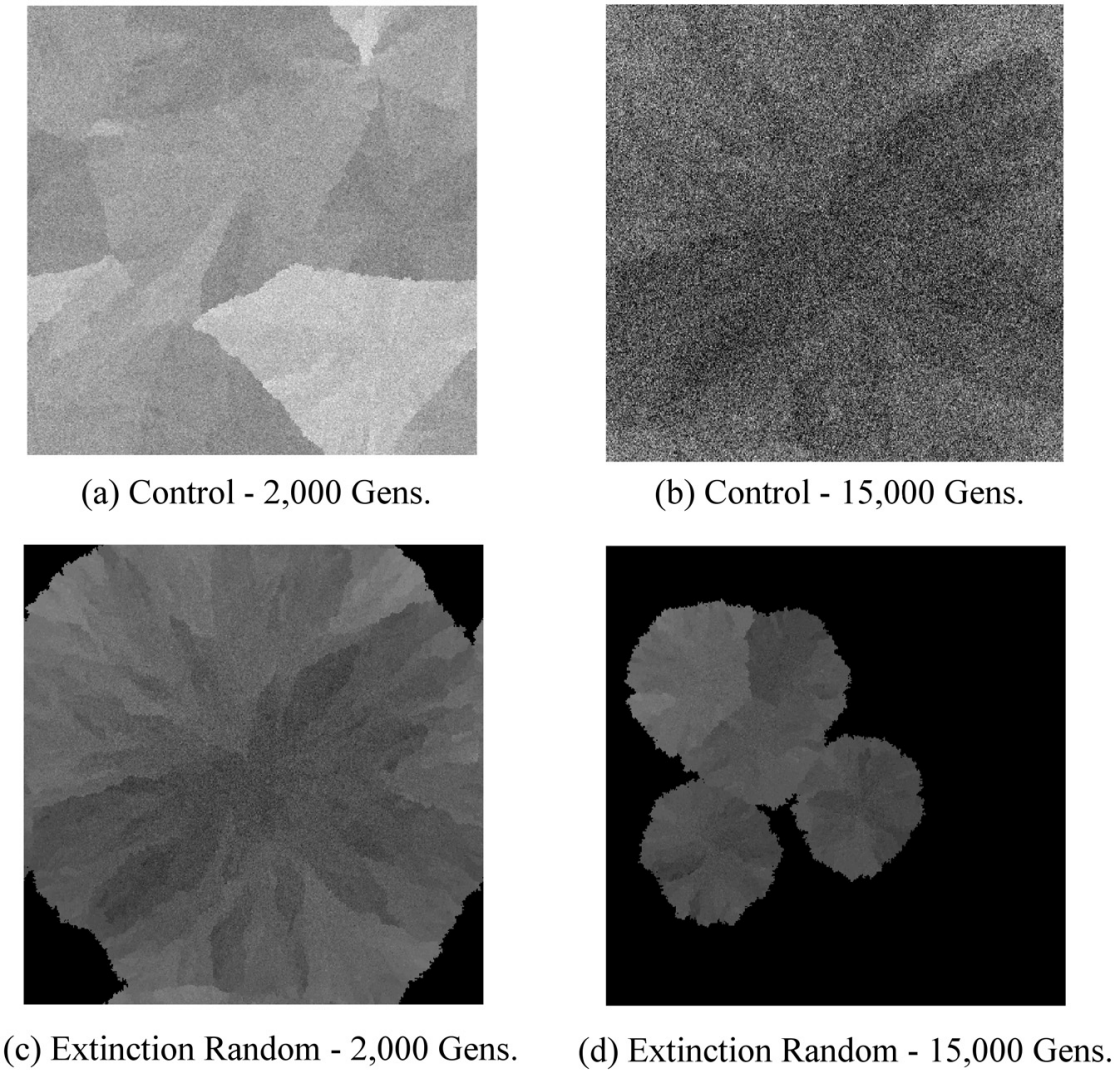
Representative evolvability snapshots in the abstract model. A heatmap of evolvability over the space of niches is shown for representative runs of the Control and Extinction Random conditions at 2,000 and 15,0000 generations. Lighter shades indicate higher evolvability, with the same scale across all four snapshots. By 2,000 generations, the Control condition has nearly saturated all the niches (a). From the saturation point to the 15,000 generation snapshot (b), the Control condition evolves only through drift. In contrast, in this particular run of the Extinction Random condition, at 2,000 generations (c), evolution can be seen recovering from an extinction event. In particular, five clusters of organisms are spreading at different speeds (relative to their evolvability) through the space of niches. In this way more evolvable lineages will spread through more niches and will be more likely to persist across extinctions. At 15,000 generations (d), the Extinction Random condition exhibits higher evolvability than the Control. The conclusion is that extinction events lead to higher evolvability.

Supporting this paper’s hypothesis, without extinction events (i.e. in the Control condition) the average evolvability of organisms reaches a plateau after all niches become saturated. While *maximum* evolvability of organisms may still rise from the resulting genetic drift in such saturated niches [13], there is no overall bias towards an increase. In contrast, in all of the other conditions, average evolvability continues to increase over generations (Fig 1). The more frequent the extinction events, the higher the final evolvability (note that the Extinction Random condition has an effective interval of 2500 generations and its evolvability, correspondingly, is bounded between the Extinction 2000 and Extinction 4000 conditions). The mechanism by which evolvability increases is illustrated in Fig 2. While in Control it is mostly due to drift, with extinction events it is driven by surviving niches with higher evolvability.

During early generations in all conditions, evolvability of organisms is correlated with distance from the initial starting niche, as evolvable organisms more quickly spread through niches. However, only in the Control condition does this correlation persist throughout the simulation (*r* = 0.380, *p* < 0.001; Spearman’s r, test run on the aggregated final populations of the Control condition). In contrast, extinction events break this regularity by introducing evolutionary bottlenecks that are randomly distributed over the geometry of niches, resulting in only slight correlation between evolvability and distance from the initial niche at the simulation’s completion (for Extinction 2000, *r* = −0.0565, *p* < 0.001; Spearman’s r, other Extinction conditions are similar).

Overall, results in the abstract model show that extinction events results in higher evolvability in the final population. Next, this idea is demonstrated in a more concrete model evolving behaviors for simulated robots.

### The evolutionary robotics model

Supplementing the abstract computational evidence provided by the previous section, a more concrete model is considered in this section. There are two main motivations. First, while informative, the abstract model is minimal by design: A genotype consists of three numbers that represent abstract properties of phenotypes directly. However, in nature such properties instead emerge indirectly from the genotype to phenotype mapping, which depends upon a number of complex and inter-related processes (e.g. protein interactions, development, and an organism’s environment). In particular, a significant factor is the ability of the genotype to phenotype mapping itself to evolve [24]. Thus a model with a genotype to phenotype mapping that is more *grounded* (i.e. behavior and evolvability emerge from a physical simulation of what the genotype encodes) and more *flexible* (i.e. some aspects of the genotype to phenotype mapping can evolve), can help probe whether the results of the abstract model generalize.

Second, artificial instantiations of evolutionary systems also have practical engineering applications [25, 26]. For this reason, evolvability is a property of interest also to computer scientists who study and apply evolutionary algorithms [24], particularly because artificial systems are known to lack evolvability [24, 27]. Thus, the conclusions from the abstract model may be useful for artificial evolution. Inversely, if extinction events improve evolvability in artificial evolution, it can be seen as computational evidence that they may play a similar role in biology.

The field of evolutionary robotics (ER; [20]) provides models that can be leveraged for both of these purposes. ER is motivated on one hand by biological evolution’s ability to create complex and robust autonomous agents, and on the other by the difficulty in hand-programming robots to achieve such behaviors. The central idea is to apply artificial evolution to construct behaviors and morphologies for robots [25, 28, 29].

The ER tasks employed in this paper are typical in ER research and are adapted from previous experiments [13, 30, 31]. In both tasks, the morphology and environment of the robot are held constant; the *controllers* are evolved to specify how a robot will behave, i.e. how it reacts to information from on-board sensors such as cameras or range-finders. In particular, evolution acts upon genotypes that specify the construction of artificial neural networks (ANNs), which provide an effective way to represent complex relationships between sensors and effectors.

The genotype-to-phenotype mapping embeds one such ANN into a simulated robot to act as a closed-loop controller. Many times per second the robot’s sensory information is provided as input to the ANN, and the resulting output signals from the ANN control the robot’s motors. The resulting behavior of the robot interacting with its environment is considered the phenotype. Thus in this model a genotype ultimately maps to the behavior of a robot, which is grounded by the physics of the simulation. Additionally, mutation rates can themselves evolve [27], providing flexibility in the genotype-to-phenotype mapping; such changes in mutational rate have been linked to evolvability in both biological and artificial systems [32, 33].

The first ER task consists of a simulated wheeled robot embedded in a maze, while the second focuses on an unstable bipedal robot. While other ER tasks could be applied, the chosen two are well-understood [30, 31, 34], span two distinct classes of locomoting robots, and provide a significant challenge for evolution.

Evolution proceeds in the ER model as it does in the abstract model, with separate populations subsisting on distinct and limited resources. In contrast to the abstract model, where an individual’s niche is specified directly in its genotype, the ER model defines niches based on the robot’s *behavior*. The niches thus stand for behavioral specializations. They are called niches to be consistent with the abstract model and to make a high-level analogy to biology transparent. They create a a space of possibilities that incentivizes discovering and sustaining many different behaviors, and in that sense can be seen as an abstraction of Hutchinsonian niches [35] with complete competitive exclusion [36].

In both tasks, a space of behavior specializations is constructed by imposing a uniform grid over the ground plane of the simulation; an organism’s niche is determined by the grid square it occupies at the simulation’s conclusion. The idea is that different phenotypes may affect niching more than genotypes or neural structures (e.g. changes in beak size and preferences enabled the specialization of Galapagos island finches [37]). Thus many different genotypes encoding many different behaviors may all be mapped to the same niche. This kind of space is consistent with Hutchison’s conception of niches as n-dimensional hypervolumes [35], and has been utilized in previous studies [13, 27]: Each grid square corresponds to a resource that supports up to a certain number of organisms with similar behavior.

By providing a complex mapping from genotype to behavior, ER genotype-phenotype maps require *empirical* evolvability measures. That is, to estimate evolvability, it is necessary to explore the distribution of the behaviors of an individual’s offspring, which requires evaluating a sample of such offspring in a physical simulation. In this way, ER is similar to biological evolution, where predicting evolvability from an organism’s genotype alone is generally intractable. Thus, in contrast to the abstract model, evolvability in ER is an emergent property instead of merely a theoretical construct: A more evolvable organism in the ER model leads to more diverse phenotypes through mutation. Thus, evolvability is estimated by counting the number of distinct behavioral specializations that result from mutating the organism (details of this measure can be found in the Methods Section).

In the ER experiments, five conditions parallel to those applied in the abstract model are compared. Because the simulations are much more demanding computationally, they are run for fewer generations and extinction events take place on a correspondingly quickened scale. Preliminary experimentation showed that although the results tend to be more noisy, the setup is valid, providing sufficient time for behavioral specializations to saturate inbetween extinctions. The *Extinction 300*, *Extinction 600*, and *Extinction 900* conditions impose extinction events at regular intervals of 300, 600, 900 generations, respectively, and the *Extinction Random* condition imposes them at random intervals chosen uniformly between 300 and 900 generations. Extinctions are implemented in the same way as in the abstract model (i.e. only five niches are spared), and the Control condition remains the same (i.e. it does not include any extinction events).

**Fig 3:**
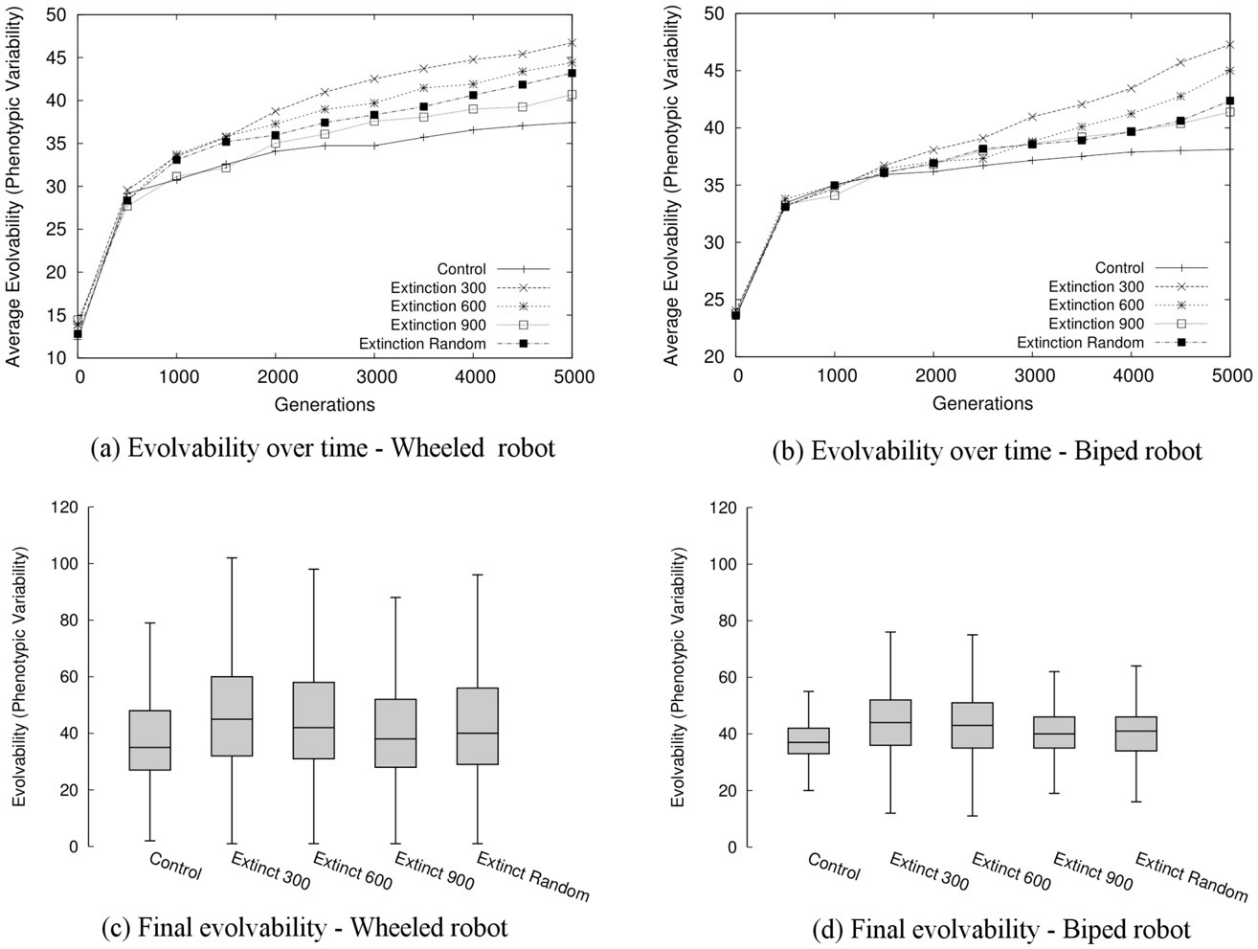
Evolvability in the evolutionary robotics model. The average evolvability of the population over generations of evolution is shown for the (a) wheeled robot and (b) biped robot tasks, averaged first over the population, and then over 60 independent runs. Relative to the Control condition, in all pair-wise comparisons populations in each of the Extinction conditions demonstrate significantly higher average evolvability (Mann-Whitney U test; *p* < 0.05), and variance in evolvability (Fligner’s test; *p* < 0.05) by 5000 generations. Figs (c) and (d) illustrate how evolvability is distributed in final populations; the evolvability of individuals in final populations is aggregated over all independent runs for each condition (bands in the boxes divide quartiles, and whiskers denote 1.5 times IQR). The conclusion is that extinction events tend to increase both evolvability.

**Fig 4:**
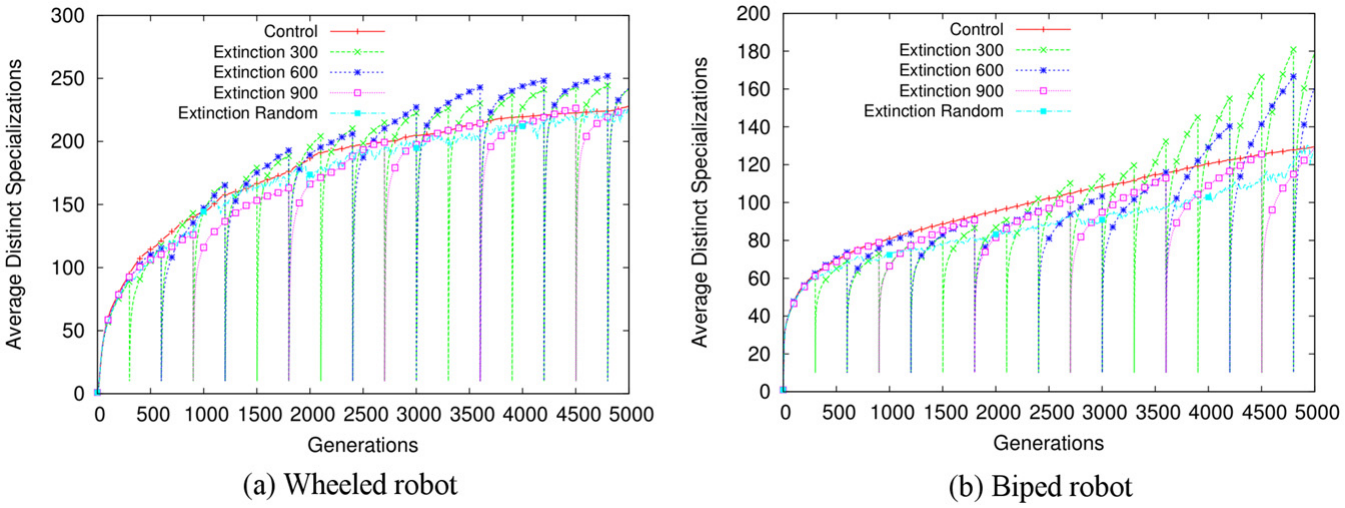
Behavioral diversity in the evolutionary robotics model. The dynamics of how specializations (i.e. niches) are occupied over generations is shown for the (a) wheeled robot and (b) biped robot tasks, averaged over 60 independent evolutionary runs. The Control condition populates monotonically more specializations over evolution because it is not subject to extinction events. In contrast, in the Extinction conditions extinctions decimate the population at regular intervals. The individuals left untouched in a few specializations then repopulate the vacated ones. Lineages with greater phenotypic variability (i.e. greater evolvability) spread through more niches and thus are more likely to persist through extinctions. Accordingly, the Extinction conditions rebound increasingly quickly after each extinction event, indicating that later generations are more evolvable. In some conditions (e.g. the Extinction 600 in the wheeled robot task), despite regular decimation, the number of specializations eventually becomes greater than in the Control condition. (Note that when extinctions occur at random intervals, averaging obfuscates this dynamic. However, S2 Fig shows that individual Extinction Random runs indeed proceed through similar accelerating rebounds as the other Extinction conditions.

**Fig 5:**
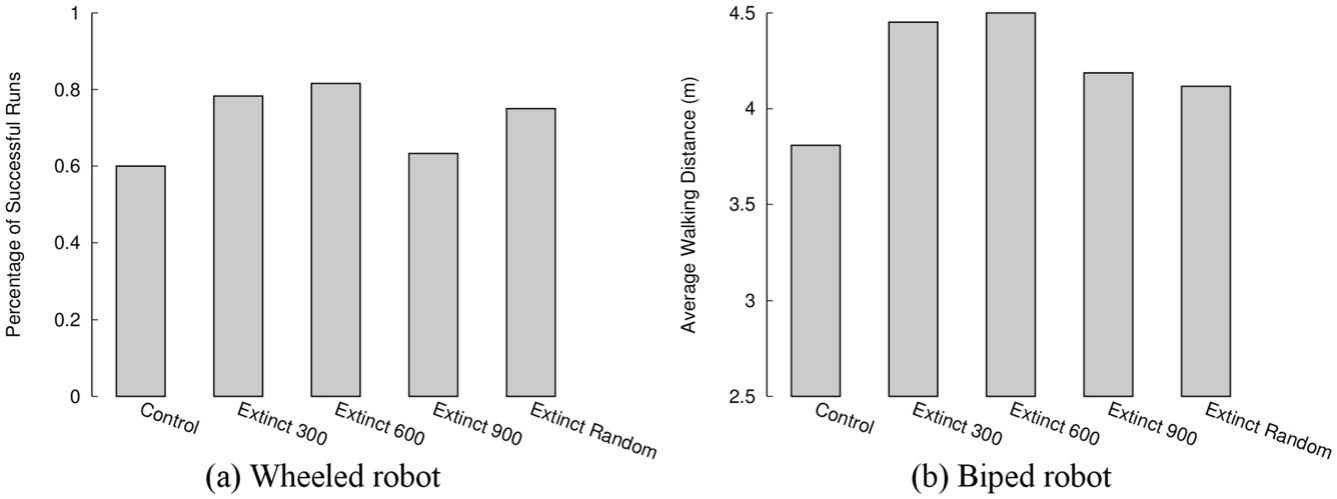
Evolutionary success in the evolutionary robotics model. The ability of evolution to generate well-adapted solutions is shown for the (a) wheeled robot and (b) biped robot tasks. In the (a) wheeled robot task, a successful robot can navigate the full extent of the maze. In the (b) biped robot task, a successful robot can walk a long distance. Pair-wise comparisons of average performance show that the Control condition never significantly outperforms any of the Extinction conditions, while the Extinction 600 condition significantly outperforms the Control condition in both domains (Mann-Whitney U-test for biped comparisons, Fisher’s exact test for maze comparisons; *p* < 0.05). Extinction events thus improve the final performance in both tasks.

As in the abstract model, evolvability in the Extinction conditions increases significantly relative to the Control condition in both tasks (Fig 3). Fig 4 visualizes the behavioral diversity over this process. Each extinction event is followed by a rebound that is more diverse than the previous one. Thus, extinction events result in indirect selection for more evolvable individuals. Further, Fig 5 illustrates that evolution with extinction events results in more successful final evolved controllers (see also S3 Fig, which highlights the differences in performance between the best policies found over all independent runs of each condition). Thus the overall conclusion is that by including extinction events in the ER models, evolution is likely to discover evolvable and effective individuals.

## Conclusions

The computational experiments in this paper provide evidence for the hypothesis that extinction events accelerate evolution. In an idealized abstract model as well as in a complex concrete ER model, extinction events led to increased evolvability.

These results reinforce a positive interpretation of how extinction events impact evolution [5, 8]. While superficially extinction events appear to disturb biological evolution [38], such wanton destruction may yet benefit the process as a whole. An interesting corresponding change of perspective is that the abilities of any particular lineage to survive and reproduce are relatively unimportant to evolution’s long-term potential. This potential is more greatly facilitated by capacity to generate diverse phenotypic novelty. It is precisely this potential that extinction events accentuate to the *detriment* of specific lineages and species. As the results show, more expansive evolution can result from repeated indiscriminate extinction.

This expansive effect of extinctions can be interpreted in the context of the red queen [39, 40] and court jester [40, 41] hypotheses, i.e. the relative importance of biotic and abiotic factors in evolution. Given that the most likely causes of past severe extinction events appear to be abiotic [42, 43], and that direct biotic adaptive pressure is not included in the simulated models, one interpretation is that the models demonstrate a possible mechanism by which abiotic court jester forces alone can accelerate evolution. However, echoing previous calls for the red queen and court jester effects to be considered together [41], evolvability increases in the models through a combination of repeated cycles of abiotic extinctions followed by indirect biotic competition to refill as many vacated roles as possible. That is, a sudden abiotic shock can stage a stadium for a biotic race that culls the most evolvable survivors.

To aid interpreting the results, it is important to relate the assumptions of this paper’s models to biological reality. As a proof of concept demonstrating the possible viability of the proposed mechanism, the severity and frequency of extinctions is exaggerated to accentuate their effect. For example, it is unlikely that natural systems experience extinctions as extreme as in the ER model, i.e. imposed every 300 generations with only five distinct survivors. Computational costs in simulating the robot model prevent modeling the vast expanse of evolutionary time in which more realistic intervals of extinction could be explored; yet it is intriguing that even in such an intense form extinctions can lead to more effective robot controllers. Note also that evolvability increased significantly across the entire range of explored intervals (i.e. spanning from 300 to 900 generations); the mechanism may thus be independent of extinction intervals and may generalize to grander spans of generations. On the other hand, the particular effectiveness of the Extinction 600 condition suggests that in particular the short ER simulations may benefit from tuning the interval parameter (as is ubiquitous in machine learning [44, 45]). In addition, because geography is not modeled, no particular spatial scale is directly implied. The model is inspired by large-scale extinction events but the proposed mechanism may act on multiple spatial scales, i.e. globally when extinctions events are global, or within local ecosystems when extinctions are similarly local.

It may also be informative for future studies to probe the simplifying assumptions of the model. For example, extinctions are effectively random on the level of niches (or specializations) instead of being selective but unpredictable [5] as is suggested by biological evidence, and do not depend on effective population size. However, the results are unchanged even when extinctions are less severe (S1 Fig) and less regular (the Extinction Random condition), which suggests that the effect is robust.

Finally, the conclusions of this study may provide insight into similar phenomena in other domains, such as creative destruction in business [46] and the way wildfires help renew ecosystems [47]. In such cases, the temporary effect of a mechanism is superficially destructive to particular participants in a process, while the ultimate longer-term effect is to make the process as a whole more innovative and robust. This deceptive pattern seems to be often exploited by evolutionary systems in general, and thus it may be useful to consider other seemingly destructive forces in the evolution of economies, products, and ideas in the same light.

## Methods

The following sections provide more details on the computational models used in the experiments in this paper.

### Abstract Model Details

The space of niches is defined as a 401 x 401 toroidal grid. Evolution was seeded with an initial individual at the center square with evolvability initialized to 0.025. At the beginning of each generation, an individual’s niche was perturbed with a probability equivalent to its evolvability, and its evolvability itself was perturbed with a fixed probability of 0.005. Changes in evolvability were drawn from a uniform distribution within [−0.0025, 0.0025]. In the simulation, if a niche becomes full after individuals *within* that niche reproduce, then there is no chance for individuals from an outside niche to mutate into it. This restriction codifies the assumption that there are often significant barriers to invading an occupied niche [6]. When an extinction event is simulated, five occupied niches are randomly selected to be spared; individuals in all other niches are discarded.

### Evolutionary Robotics Model Details

NEAT, a well-studied algorithm for evolving neural network controllers [48], is used as the evolutionary mechanism. It allows for evolved ANNs to become more complex as evolution progresses. Note that for both evolved robots experiments, NEAT is augmented with self-adaptive mutation because it was found to result in greater evolvability to evolve, as in prior work [27].

In this model, the evolvability of a genome is calculated by counting the unique number of niches encoded by genomes in its mutational neighborhood. However, because *all* reachable mutations cannot be feasibly enumerated, instead 200 mutations of a given genome are randomly sampled to generate a reasonable *estimate* of its relative evolvability.

In both ER experiments, extinction events are implemented in the same way as in the abstract model, i.e. all individuals in five randomly-chosen occupied niches survive the extinction while all other individuals are discarded.

#### Wheeled Robot Experiment

**Fig 6:**
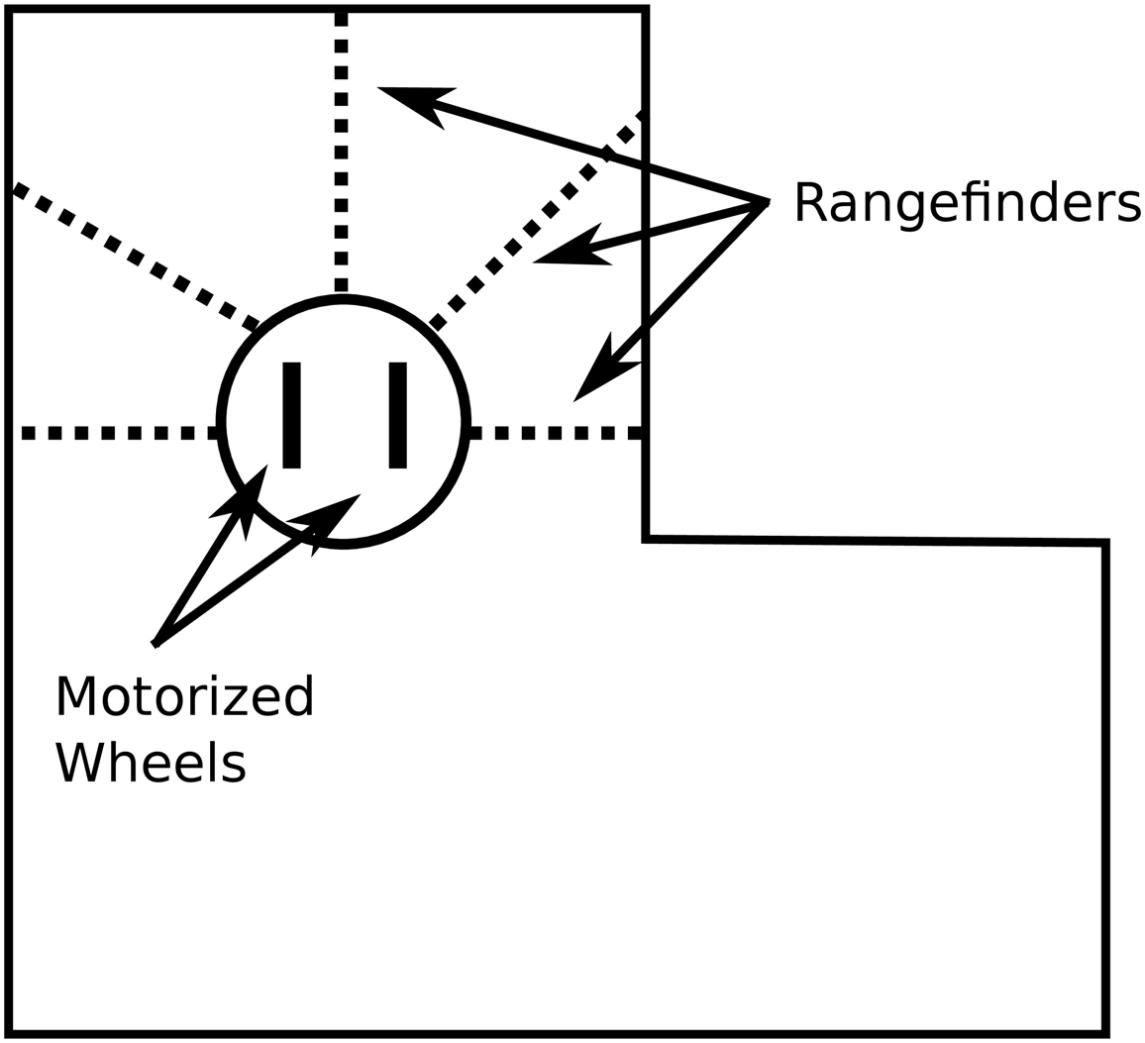
Simulated mobile robot used in the maze navigation experiment. Rangefinder sensors allow the robot to perceive obstacles, and the motors controlling its wheels enable the robot to traverse its environment.

**Fig 7:**
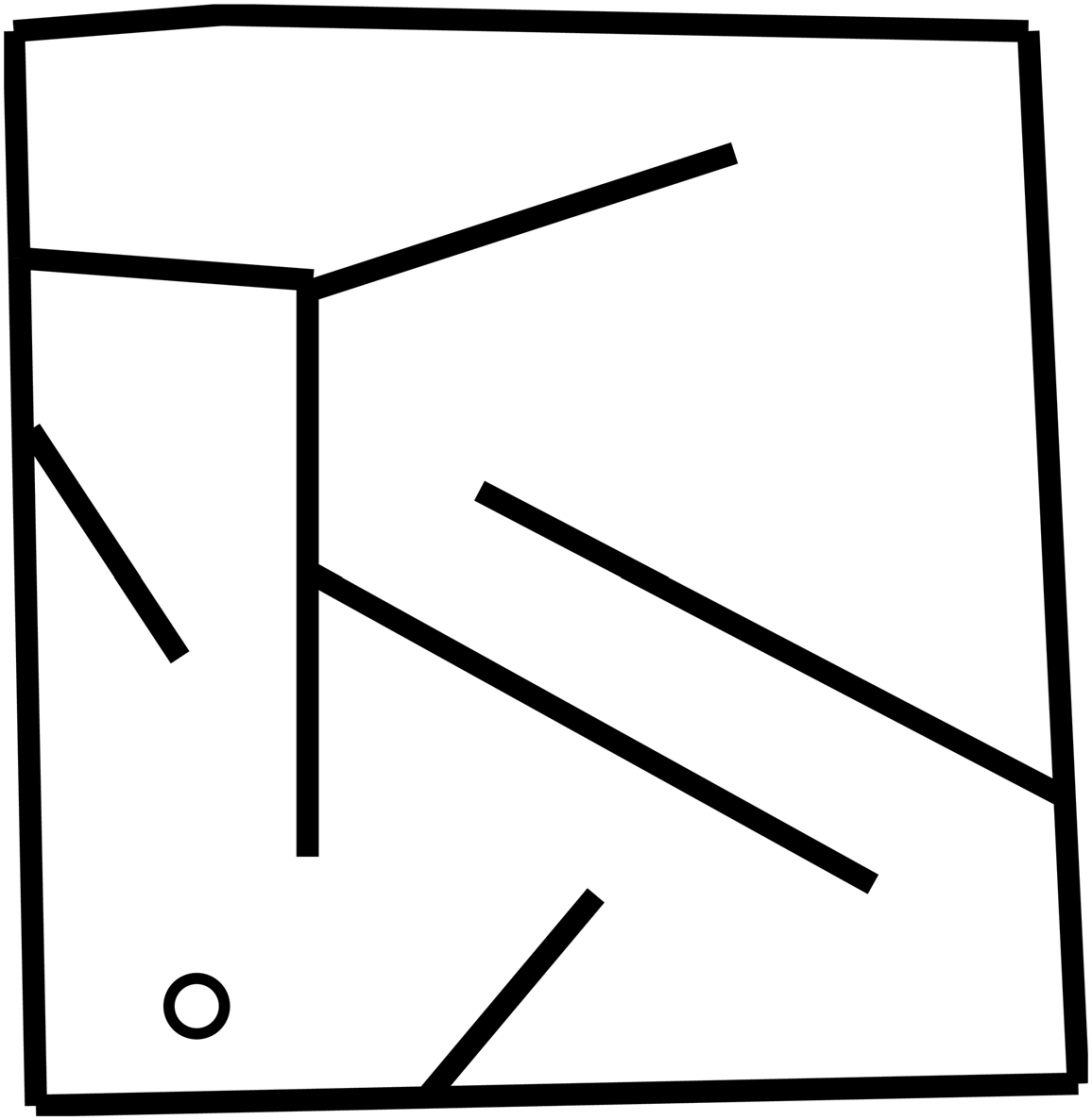
Top-down view of the maze navigated by robots in the maze navigation experiment. The circle indicates where a robot begins its trial. The trial is considered successful if the robot travels to the top left corner within 400 simulated timesteps. This particular maze is used because it is well studied [13, 27, 30, 31] and has been found to provide potential for interesting and diverse behaviors to evolve.

**Fig 8:**
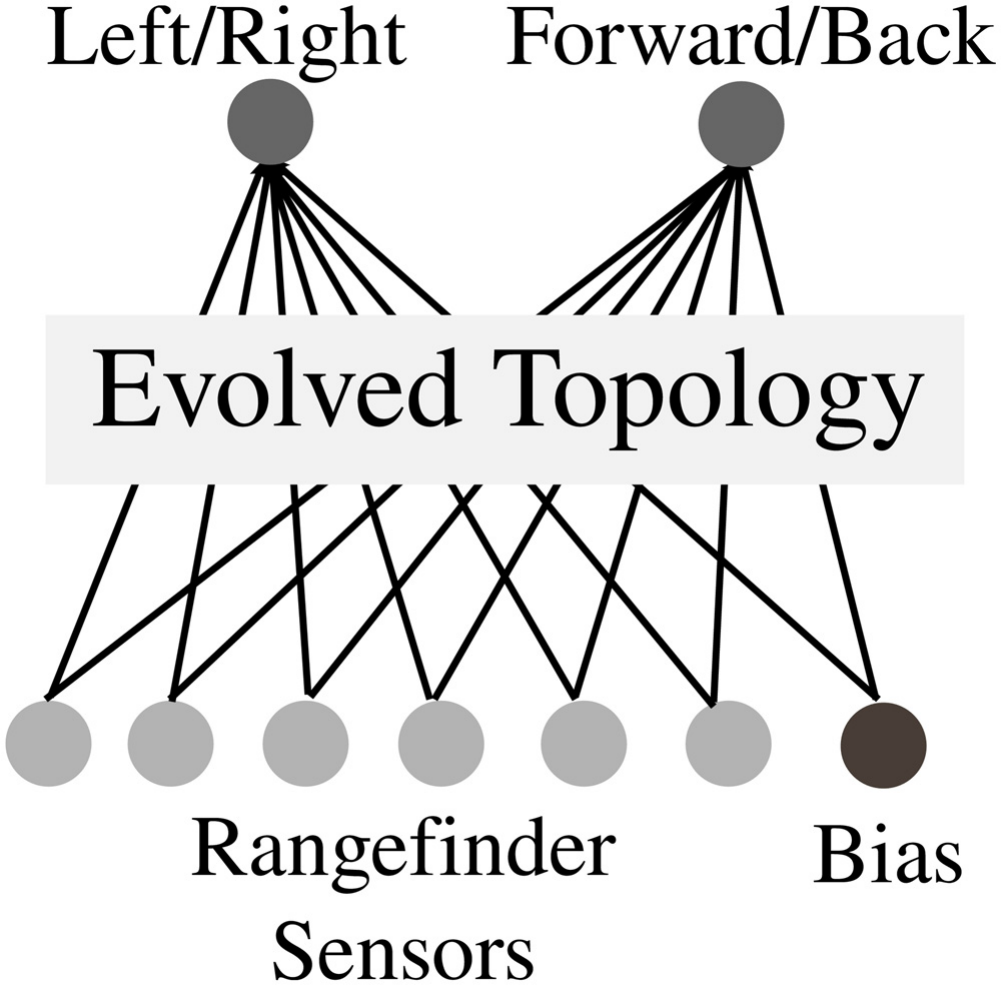
The evolved ANN controlling the maze robot. The initial topology is a fully connected network with no hidden nodes. Topologies change during evolution through structural mutations that add new nodes and connections. Connection weights are perturbed through continuous-valued weight mutations; the weights are capped between −3.0 and 3.0.

In the maze navigation experiment, a genome that maps to an ANN controls a simulated wheeled robot (Fig 6) with rangefinder sensors (Fig 7). The experimental setup follows prior work [13, 27, 30]. In particular, the fragile hard maze of Lehman and Stanley [27] is used as the environment. The initial NEAT network topology is shown in Fig 8. All NEAT parameters are adopted from Lehman and Stanley [27].

To calculate a robot’s behavioral specialization, a uniform 20 × 20 grid was superimposed over the maze. A robot’s niche (applied for measuring evolvability as well as to limit population growth) was determined by the grid square that the robot occupies at the termination of an evaluation.

#### Biped Robot Experiment

**Fig 9:**
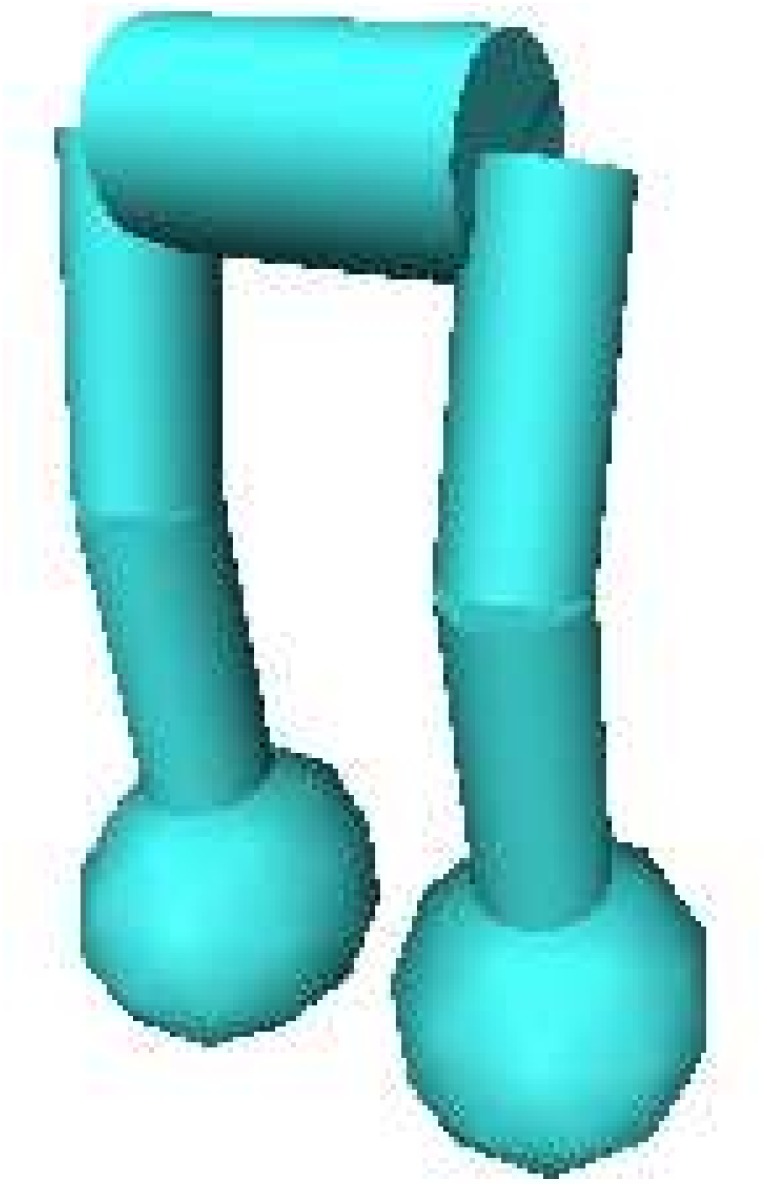
The simulated robot used in the biped experiment. The robot has motors that apply forces to achieve the joint angles that are output by the ANN. The controller has the challenging task of keeping the robot from falling while traveling as far from the starting point as possible.

In the biped robot experiment, an evolved ANN controls a three-dimensional biped robot (Fig 9) in a realistic physics simulation. Similar to the maze navigation experiment, the behavioral niches were defined based on a uniform 40 × 40 grid superimposed over an 16 meter × 16 meter portion of the environment. The robot begins the simulation standing upright at the center of the grid and the simulation proceeds until the robot falls, or until 15 seconds have passed. The grid square within which the robot’s center of gravity is contained at the end of the simulation is considered the robot’s behavioral niche. If a robot travels outside the bounds of the grid it is mapped to the nearest valid grid square.

**Fig 10:**
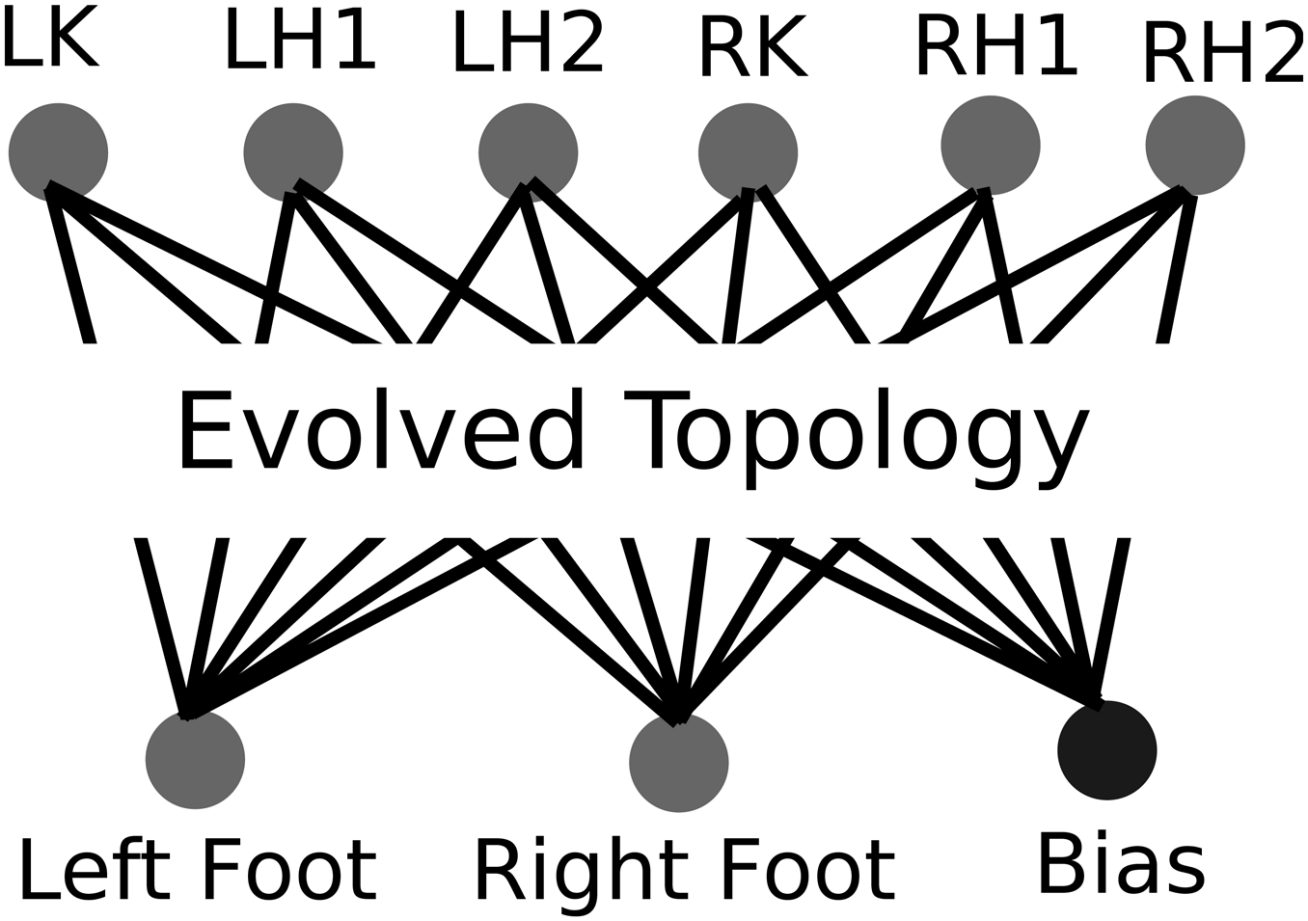
The evolved ANN controlling the biped robot. As in the maze experiment, the initial topology is a fully connected network with no hidden nodes and evolves through structural mutations throughout evolution. The ANN outputs control the motors for each of the robot’s six degrees of freedom: one in both its left and right knees (LK and RK), and two in each hip (LH1, LH2, RH1, and RH2). The ANN receives as input whether each of its feet touch the ground. As in the maze experiment, evolution discovers ANNs that achieve high fitness in the task.

All NEAT parameters were adopted from the biped walking experiment of Lehman and Stanley [27]. The initial NEAT ANN topology is shown in Fig 10.

## Supporting Information

S1 Fig Varying the severity of extinction events in the abstract model. The average evolvability of individuals (averaged over 20 additional independent runs) is shown for the Extinction 1000 and the Control condition with varying severity of extinction. Evolvability increases most when extinctions are most severe (i.e. when only five niches survive each extinction), but even with less severe extinctions, evolvability is significantly higher than in the Control condition by the end of evolution (Mann-Whitney U-test;*p* < 0.05). The conclusion is that the severity of extinctions influences the magnitude of increase in evolvability, but not the trend.
(TIF)

S2 Fig Accelerating the rebounds from extinctions. The ability of evolution to rebound following extinction events is shown for the (a) wheeled robot and (b) biped robot models. Each point indicates both the generation (x axis) at which an extinction took place, and the magnitude of the immediate rebound that follows (y axis) in each individual run of evolution. Rebound is measured by how many additional behavioral niches are occupied ten generations after the extinction event. For each model and condition including extinctions, Spearman’s correlation coefficient indicates that rebounds become larger over generations (*p* < 0.0001 for all 8 individual tests; *r* > 0.5 for all tests). The conclusion is that extinction events indirectly select for the ability to rebound, which is an intuitive proxy for evolvability.
(TIF)

S3 Fig Comparison of distance traveled by best walking policies. Performance of the best walking policies are shown for the Control condition and variations of the Extinction condition. Each Extinction condition discovers a walker that outperforms the Control condition by a significant margin, with the Extinction 300 condition discovering an overall-best policy; it walks almost twice as far as the best one over all runs of the Control condition. This result highlights the magnitude of possible gains from incorporating extinction events into evolution in engineering problems.
(TIF)
