# Supplementary figures and images for: Extinction Events Can Accelerate Evolution
Source: PLoS One. 2015 Aug 12;10(8):e0132886. doi: 10.1371/journal.pone.0132886 (PMC4533974; doi:10.1371/journal.pone.0132886)

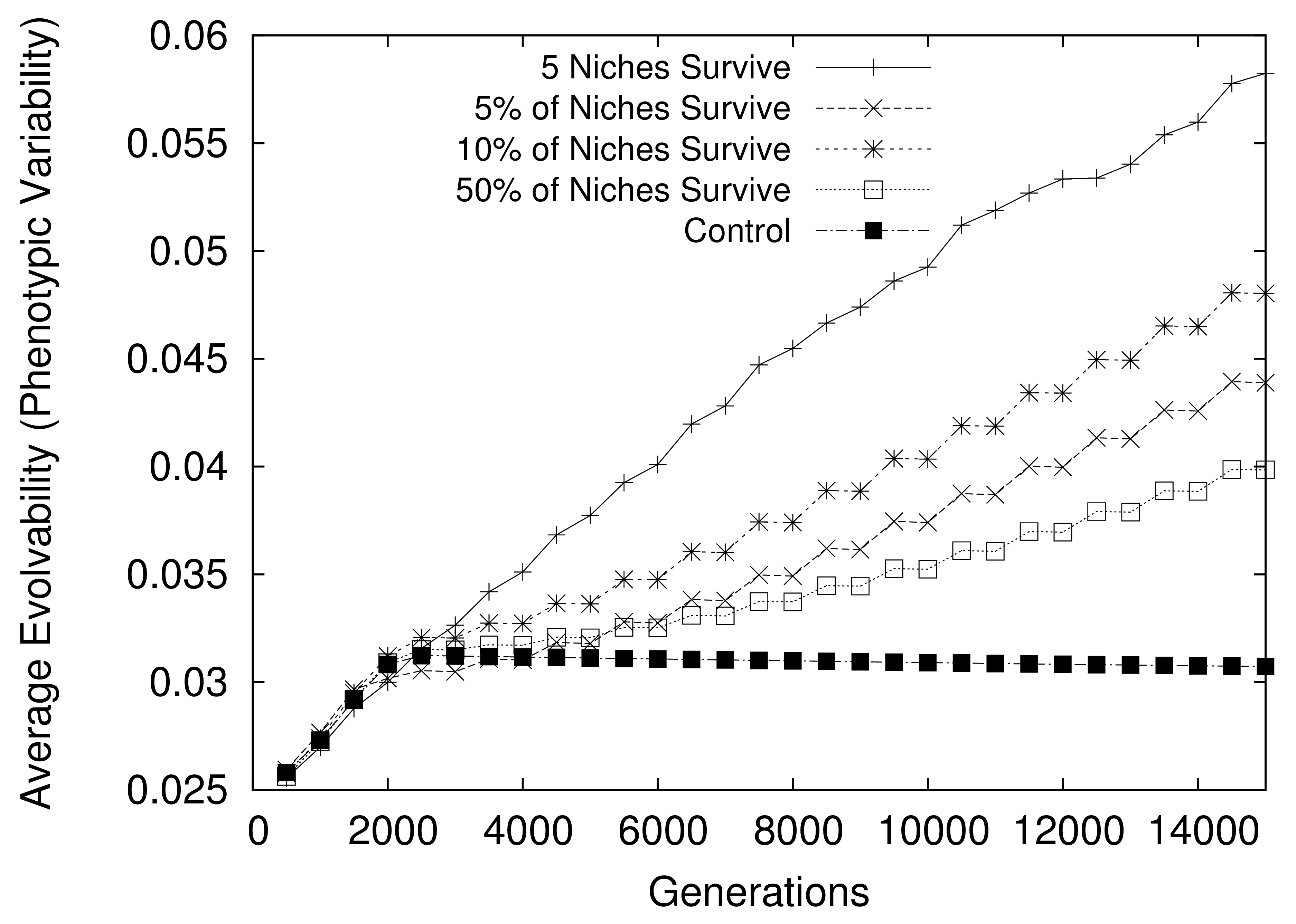

Supplement: S1 Fig — The average evolvability of individuals (averaged over 20 additional independent runs) is shown for the Extinction 1000 and the Control condition with varying severity of extinction. Evolvability increases most when extinctions are most severe (i.e. when only five niches survive each extinction), but even with less severe extinctions, evolvability is significantly higher than in the Control condition by the end of evolution (Mann-Whitney U-test;p < 0.05). The conclusion is that the severity of extinctions influences the magnitude of increase in evolvability, but not the trend. (TIF) [file pone.0132886.s001.tif]

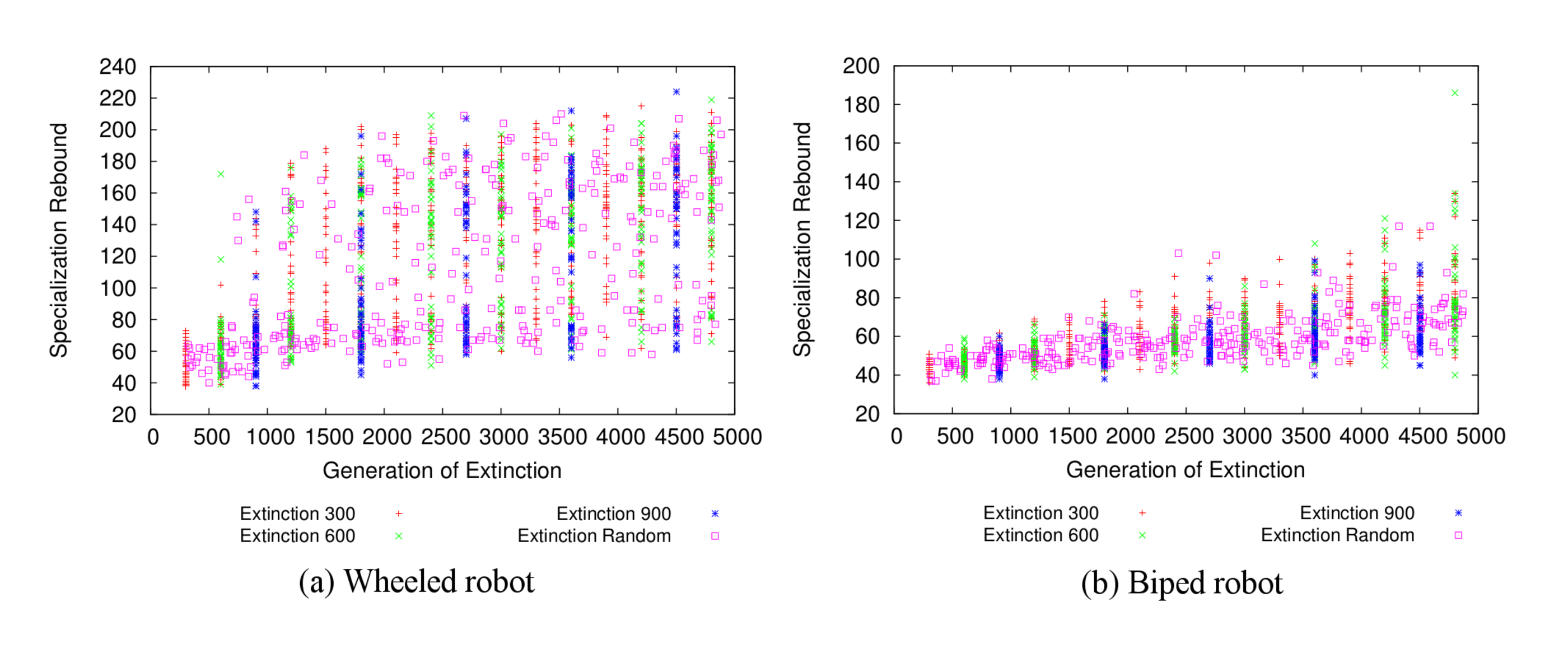

Supplement: S2 Fig — The ability of evolution to rebound following extinction events is shown for the (a) wheeled robot and (b) biped robot models. Each point indicates both the generation (x axis) at which an extinction took place, and the magnitude of the immediate rebound that follows (y axis) in each individual run of evolution. Rebound is measured by how many additional behavioral niches are occupied ten generations after the extinction event. For each model and condition including extinctions, Spearman’s correlation coefficient indicates that rebounds become larger over generations (p < 0.0001 for all 8 individual tests; r > 0.5 for all tests). The conclusion is that extinction events indirectly select for the ability to rebound, which is an intuitive proxy for evolvability. (TIF) [file pone.0132886.s002.tif]

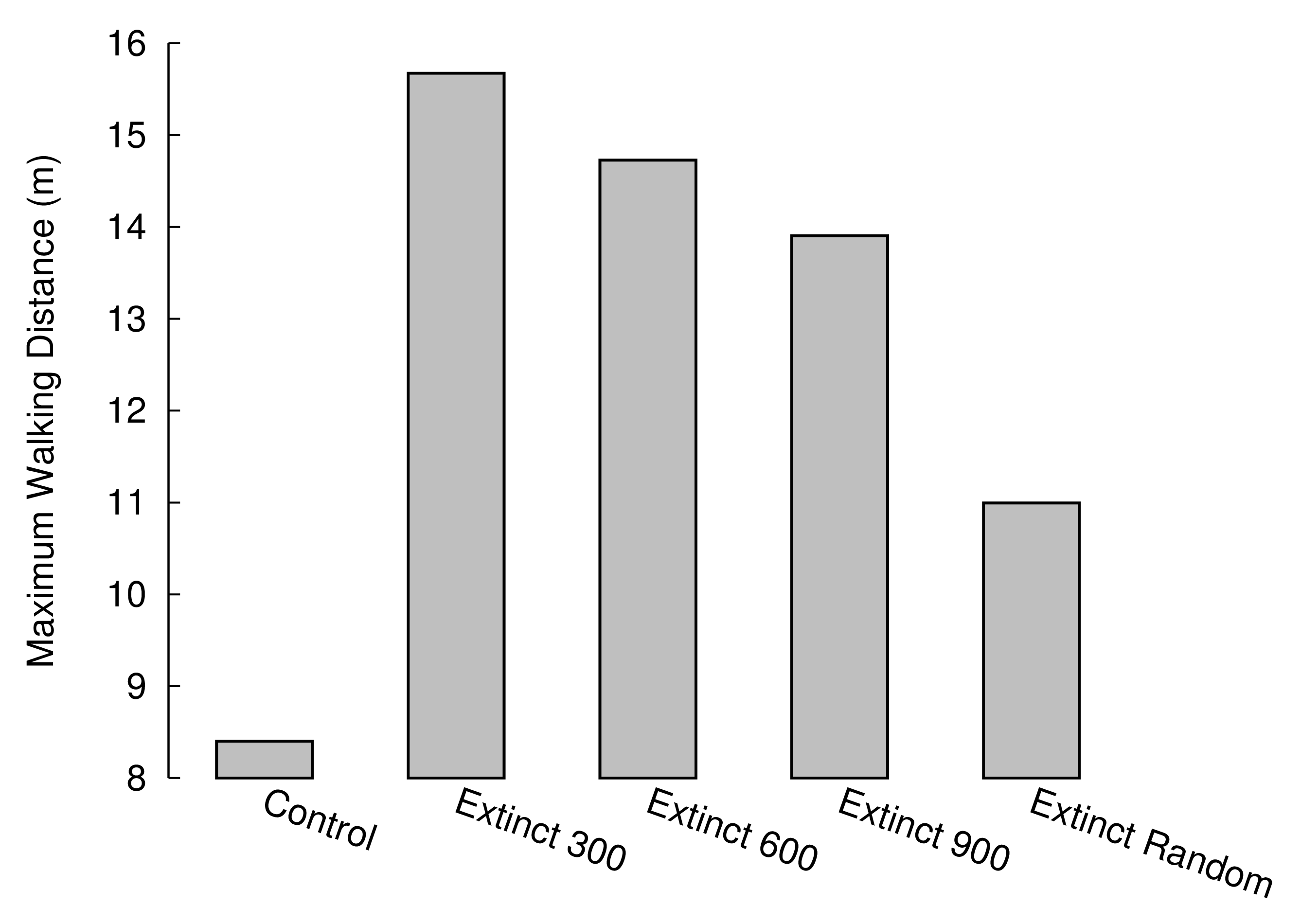

Supplement: S3 Fig — Performance of the best walking policies are shown for the Control condition and variations of the Extinction condition. Each Extinction condition discovers a walker that outperforms the Control condition by a significant margin, with the Extinction 300 condition discovering an overall-best policy; it walks almost twice as far as the best one over all runs of the Control condition. This result highlights the magnitude of possible gains from incorporating extinction events into evolution in engineering problems. (TIF) [file pone.0132886.s003.tif]
